# Variations in apolipoprotein D and sigma non-opioid intracellular receptor 1 genes with relation to risk, severity and outcome of ischemic stroke

**DOI:** 10.1186/s12883-014-0191-2

**Published:** 2014-09-28

**Authors:** Håkan Lövkvist, Ann-Cathrin Jönsson, Holger Luthman, Katarina Jood, Christina Jern, Tadeusz Wieloch, Arne Lindgren

**Affiliations:** Department of Clinical Sciences Lund, Neurology, Lund University, Lund, Sweden; Department of Neurology and Rehabilitation Medicine, Neurology, Skåne University Hospital, Lund, Sweden; Department of Health Sciences, Lund University, Lund, Sweden; Department of Clinical Sciences Malmö, Medical Genetics, Lund University, Malmö, Sweden; Department of Clinical Neuroscience and Rehabilitation, The Sahlgrenska Academy at University of Gothenburg, Institute of Neuroscience and Physiology, Gothenburg, Sweden; Department of Neurosurgery, Laboratory for Experimental Brain Research, Lund University, Lund, Sweden; R&D Centre Skåne, Skåne University Hospital, SE-221 85 Lund, Sweden

**Keywords:** Stroke, Genetics, *APOD*, *SIGMAR1*, mRS, NIHSS

## Abstract

**Background:**

In experimental studies, the apolipoprotein D (APOD) and the sigma receptor type 1 (SIGMAR1) have been related to processes of brain damage, repair and plasticity.

**Methods:**

We examined blood samples from 3081 ischemic stroke (IS) patients and 1595 control subjects regarding 10 single nucleotide polymorphisms (SNPs) in the *APOD* (chromosomal location 3q29) and *SIGMAR1* (chromosomal location 9p13) genes to find possible associations with IS risk, IS severity (NIHSS-score) and recovery after IS (modified Rankin Scale, mRS, at 90 days). Simple/multiple logistic regression and Spearman’s rho were utilized for the analyses.

**Results:**

Among the SNPs analyzed, rs7659 within the *APOD* gene showed a possible association with stroke risk (OR = 1.12; 95% CI: 1.01-1.25; *P* = 0.029) and stroke severity (NIHSS ≥ 16) (OR = 0.70; 95% CI: 0.54-0.92; *P* = 0.009) when controlling for age, sex and vascular risk factors for stroke. No SNP showed an association with stroke recovery (mRS).

**Conclusions:**

We conclude that the SNP rs7659 within the *APOD* gene might be related to risk and severity of ischemic stroke in patients.

## Background

Stroke is a major cause of death and the main cause of adult disability. Approximately 20 to 30% of all ischemic stroke patients die in the acute stages of the stroke episode while more than one third of those who survive remain dependent of daily next-of-kin support or community care six months after stroke onset [1,2]. Still, five years after stroke onset, two thirds of the survivors have some neurologic impairment and disability [3]. Recanalization of occluded vessels after embolic stroke is the only therapeutic intervention available to treat acute ischemic stroke (IS), while no pharmacological treatment that stimulate brain repair or plasticity and that might enhance recovery of lost function is at hand. However, rehabilitative training such as task-oriented practice [4] and long-term progressive resistance training [5], may enhance recovery of lost brain functions.

The multifactorial and complex features of stroke impose a considerable challenge for the understanding of the pathology and for the development of new therapies. Multiple environmental factors including co-morbidities increase the risk of stroke [6]. Likewise, stroke severity is dependent on the type of stroke, density of ischemia and duration of vessel occlusion, and is also influenced by several toxic mechanisms, most identified in experimental animal models of stroke [7]. Finally, brain repair involves mechanisms differentially activated in time and space, and include inflammation, brain remodelling and relearning of activated neural networks [8,9]. Genetic factors influence the impact of these innate cellular mechanisms and environmental factors, affecting risk for stroke, as well as the severity of brain damage and the subsequent functional outcome [10].

Previous clinical studies have shown that allelic variants within the *PDE4D* gene, chromosome 9p21 and the AB0 locus may be associated with IS risk [11-13]. Also variants in *HDAC9*, as well as in chromosome 6p21.1 and 9p21, have shown association with large vessel IS [14-17], and variants in *PITX2* and *ZFXH3* may affect cardioembolic stroke risk according to other studies made [18,19]. In contrast to the situation regarding IS risk, reports on genetic factors contributing to outcome after stroke are scarce. However, a study has reported that the apolipoprotein E (APOE) ε2 polymorphism might contribute to variability in outcomes after hemorrhagic stroke [20]. Likewise, an association was found between polymorphisms of the *COX-2* and Glycoprotein IIIa genes on functional outcome 90-days after IS [21]. These studies clearly demonstrate the potential of genetic analysis in identifying mechanisms involved in functional recovery of stroke patients. More recently, genetic variations in the human dopamine system were associated with motor learning after stroke [22]. This indicates the potential of genetic analysis in identifying relevant mechanisms involved in stroke and therapeutic targets.

The apolipoprotein D (APOD) has been suggested to be related to stroke not only by virtue of its ability to influence trafficking of lipids but also by modulating oxidative stress, synaptic plasticity and cell death [23,24]. Moreover, APOD appears to be associated with several neurological diseases and normal ageing [25], schizophrenia [26], Alzheimer's disease (AD) [27,28] and Parkinson’s disease (PD) [29]. APOD levels increase with age [30], with higher levels in women than in men [25]. Also, in experimental models of stroke [31] and trauma [32], the levels of APOD are elevated. Polymorphisms of the *APOD* gene have been associated with increased risk of AD [28,33]. The general increase of APOD levels in a broad range of disease states suggest that the protein may be induced in response to stress. Indeed, APOD appears to be an anti-oxidant [34] dependent on the integrity of the Met93 of this lipoprotein [35]. In animal models of stroke, increased APOD levels are correlated with better functional recovery, implying a possible function of APOD in the repair processes after stroke [31].

Whilst the apolipoproteins are trafficking lipids *among* cells [23], the sigma receptor type 1 (SIGMAR1, sometimes also denoted SIG1R or OPRS1) is involved in signalling and trafficking of lipids and proteins *within* cells [36]. Through these mechanisms the SIGMAR1 may modulate cell death and brain plasticity in experimental models of stroke [37]. The SIGMAR1 appears to play a central role in central nervous system (CNS) diseases since polymorphisms in the *SIGMAR1* gene are associated with depression [38], schizophrenia [39] and alcoholism [40] as well as AD [41].

With this background we aimed to investigate whether polymorphisms in the *APOD* and *SIGMAR1* genes influence stroke severity as well as functional outcome in patients suffering from IS. By including a group of control subjects we also assessed these polymorphisms’ possible impact on IS risk.

## Methods

### Study subjects

The study was approved by the ethical committee at Lund University, Lund (application 543/2008). We included 2241 consecutive first-ever IS patients of all ages from Lund Stroke Register (LSR) and 840 first-ever or recurrent IS patients below 70 years of age from the Sahlgrenska Academy Study on Ischemic Stroke (SAHLSIS), Gothenburg. Both LSR and SAHLSIS have been described previously [42,43]. Patients were included if they had clinical symptoms of IS, confirmed by CT or MR or autopsy of the brain, provided DNA for analysis, and if they or their next of kin had given informed consent to participate. Exclusively for the IS risk association assessments we also included control subjects from the same geographical areas with age and gender distribution similar to those in the IS cohort. The 1595 control subjects (929 from LSR, 666 from SAHLSIS) were randomly selected from Swedish population registers from the same areas and matched for age and gender to the patients. The SAHLSIS sample included younger participants (with range 18–69 years) than the LSR sample (with range 17 to 102 years). The proportion of men was thus larger in the SAHLSIS sample (Table 1).

**Table 1:** **Characteristics of control subjects and ischemic stroke (IS) cases**

	**LSR**		**SAHLSIS**		**Combined**	
	**Controls (N = 929)**	**IS cases (N = 2241)**	**Controls (N = 666)**	**IS cases (N = 840)**	**Controls (N = 1595)**	**IS cases (N = 3081)**
**Age**, Median (min, max)	76 (17, 96)	76 (18, 102)	58 (18, 70)	58 (18, 69)	66 (17, 96)	69 (18, 102)
**Male sex**, Number (%)	529 (57)	1169 (52)	391 (59)	550 (66)	920 (58)	1719 (56)
**Diabetes mellitus**, n	925	2139	664	840	1589	2979
Number (%)	69 (8)	550 (26)	33 (5)	153 (18)	102 (6)	703 (24)
**Hypertension**, n	925	2183	665	829	1590	3012
Number (%)	438 (47)	1479 (68)	230 (35)	485 (58)	668 (42)	1964 (65)
**Current smoking**, n	927	2193	666	836	1593	3029
Number (%)	92 (10)	423 (19)	131 (20)	323 (39)	223 (14)	746 (25)
**NIHSS at stroke onset**, n	--	1983	--	581	--	2564
0-7, Number (%)	--	1482 (75)	--	448 (77)	--	1930 (75)
8–15, Number (%)	--	338 (17)	--	92 (16)	--	430 (17)
16- , Number (%)	--	163 (8)	--	41 (7)	--	204 (7)
**mRS at 3 months**, n	--	1157	--	565	--	1722
0–2, Number (%)	--	625 (54)	--	435 (77)	--	1060 (62)
3, Number (%)	--	196 (17)	--	80 (14)	--	276 (16)
4, Number (%)	--	111 (10)	--	41 (7)	--	152 (9)
5, Number (%)	--	92 (8)	--	2 (<1)	--	94 (5)
Deceased, Number (%)	--	133 (12)	--	7 (1)	--	140 (8)

LSR = Lund Stroke Register, SAHLSIS = the Sahlgenska Academy Study on Ischaemic Stroke, mRS = modified Rankin Scale, NIHSS = NIH stroke scale, N = gross sample size, n = net sample size after removal of missing values. All percentages are based on net sample sizes.

### Definition of stroke severity and stroke recovery (outcome)

For LSR patients, initial stroke severity was assessed using the NIH stroke scale (NIHSS) in the acute phase after stroke onset [44]. For SAHLSIS patients, initial stroke severity was assessed using the Scandinavian Stroke Scale (SSS) [45]. These SSS scores were transformed to NIHSS scores through the algorithm NIHSS = 25.68-0.43*SSS [46]. A NIHSS score of 8 or above but below 16 was considered to indicate a moderately severe stroke, and a score of 16 or above was considered to indicate a severe stroke [47].

For SAHLSIS, mRS at 3 months was assessed using the original scale 0–5 at a follow-up visit with a neurologist. For LSR, stroke outcome was assessed using Riksstroke data at 3 months after stroke. We used a translation algorithm to calculate mRS grades from a set of self-reported functional outcome questions available in Riksstroke data [48]. The Riksstroke data do not distinguish between mRS-grades 0, 1 and 2. However, as mRS-grade 2 is regarded as the upper limit for independence of help/support and the patient disability information relevant for this study is provided by mRS-grades 3, 4 and 5, we merged mRS-values 0, 1 and 2 into a value of 1 [49]. In addition to the original mRS grades 0–5, we added mRS grade 6 for individuals who had died at follow up for both samples.

### Phenotypes

Definitions of intermediate phenotypes diabetes mellitus, hypertension and current smoking, and IS pathogenetic subtypes (i.e. large vessel disease, LVD; small vessel disease, SVD; and cardioembolic stroke, CE; have been described previously [11,50,51].

### Selection of genetic variants and genotyping

Seven SNPs in *APOD* and five SNPs in *SIGMAR1* (or in the immediate vicinity of these regions) were selected using two different criteria: (1) SNPs serving as markers were selected based on their low pairwise linkage disequilibrium and a population frequency of 5% or more for the two gene regions (N = 7); (2) SNPs representing non-synonymous genetic variants with low population frequency but still above 0.1% in European populations were chosen based on their probable impact on protein function (N = 6). One of these latter non-synonymous variants, rs1800866 in *SIGMAR1*, is frequent enough to also be used as a marker. The genotypings were performed at our local lab in Malmö, Sweden using Sequenom technology, except for rs76929107 at the *APOD* locus and rs1800866 at the *SIGMAR1* locus that were genotyped at LGC Genomics (former KBioscience), UK (http://www.lgcgenomics.com), using IPLEX on a MassARRAY platform (Sequenom, San Diego, CA, USA).

We scored the minor allele count of each SNP, i.e. 2, 1 or 0, and used these in additive models. Monomorphic SNPs were excluded from further analyses.

### Statistical methods

All included SNPs were tested for possible departure from Hardy-Weinberg equilibrium by chi-square test with one degree of freedom. These tests were performed on the control subjects included solely for the IS risk association analyses.

The possible association of each selected SNP with IS risk (i.e. IS patients versus control subjects) was analyzed by use of simple logistic regression, and multiple logistic regression controlling for age, gender, diabetes mellitus, hypertension and current smoking [11]. For the stroke severity response variable we used Spearman rank correlation as well as simple and logistic multiple regression with dichotomized stroke severity response (with risk category defined by NIHSS ≥ 8 and NIHSS ≥ 16, respectively) [47]. We also assessed functional outcome in a likewise manner (with risk category defined as mRS ≥ 3).

By using non-parametric statistics for the assessments of the possible impact of polymorphisms on the NIHSS and mRS scores, we were able to obtain effect measures and *P-*values that were not distorted by incorrect assumptions about these non-continuous variables.

SNP rs7659 was significantly associated with stroke severity in a first-step test. We therefore performed subsequent analyses involving subgroups including study group, gender, and age (</≥70 years) [50,52]. SPSS software (PASW/SPSS, version 18, IBM Corporation, Armonk, NY, USA) was used as a computational tool for these assessments.

## Results

### Ischemic stroke risk

Table 2 displays (1) the frequencies of all ten non-monomorphic SNPs for LSR and SAHLSIS joined together, and (2) the results of association analyses of these SNP frequencies against IS risk. All SNPs except rs11559048 conformed to the Hardy-Weinberg equilibrium criterion (Table 2). One SNP, rs7659 within the *APOD* gene region, was associated with IS risk (OR = 1.11; 95% CI: 1.01-1.22; *P* = 0.038 when tested by univariate analysis, and OR = 1.12; 95% CI: 1.01-1.25; *P* = 0.029 when using multiple logistic regression analysis controlling for covariates age, gender, diabetes mellitus, hypertension and current smoking). However, none of these *P*-values were significant when considering Bonferroni correction for multiple testing.

**Table 2:** **Analysis of association between ischemic stroke risk and ten** ***APOD*** **and** ***SIGMAR1*** **SNPs**

SNP*	**Allele pair**	**Control subj. number of genotypes**	**IS patients number of genotypes**	**Crude OR (95% CI)**	Multiple LR** OR (95% CI)
***SIGMAR1*** **:**					
rs11559048	CC	1530	2695	0.51 (0.21-1.26)	0.58 (0.91-1.20)
	CT	8	9	*P* = 0.145	*P* = 0.491
	TT	1	-		
rs1800866	TT	1128	2141	1.03 (0.92-1.16)	1.02 (0.90-1.16)
	TG	401	795	*P* = 0.615	*P* = 0.768
	GG	46	88		
rs12001648	CC	1393	2420	0.95 (0.79-1.15)	0.85 (0.70-1.04)
	CT	165	286	*P* = 0.595	*P* = 0.120
	TT	9	8		
rs7036351	GG	1130	1944	1.03 (0.91-1.16)	1.02 (0.89-1.16)
	GA	399	693	*P* = 0.675	*P* = 0.789
	AA	40	77		
rs3808873	GG	833	1475	1.00 (0.89-1.11)	0.98 (0.88-1.10)
	GA	491	887	*P* = 0.950	*P* = 0.779
	AA	91	153		
***APOD*** :***					
rs76929107	CC	1540	2943	1.06 (0.74-1.51)	1.10 (0.75-1.59)
	CT	44	91	*P* = 0.761	*P* = 0.634
	TT	1	1		
rs5952	TT	1548	2710	1.90 (0.52-6.92)	2.23 (0.57-8.75)
	TC	3	10	*P* = 0.329	*P* = 0.251
	CC	-	-		
rs34697430	GG	435	769	1.00 (0.92-1.09)	1.01 (0.92-1.11)
	GA	784	1317	*P* = 0.966	*P* = 0.895
	AA	349	623		
rs7659	AA	803	1306	1.11 (1.01-1.22)	1.12 (1.01-1.25)
	AG	617	1177	*P* = 0.038	*P* = 0.029
	GG	130	240		
rs823510	TT	880	1576	0.99 (0.89-1.09)	0.99 (0.89-1.11)
	TG	590	981	*P* = 0.805	*P* = 0.884
	GG	82	164		

*)All genotypes were conforming to the Hardy-Weinberg equilibrium criterion (with *P* = 0.093 or more when using a chi-square test on the control subjects), except for rs11559048 that showed a significant Hardy-Weinberg disequilibrium (*P* < 0.001).

**)ORs obtained by multiple logistic regression analysis controlling for covariates age, gender, diabetes mellitus, hypertension and current smoking.

***)Two additional *APOD* encoding SNPs, rs5954 and rs5955, were genotyped but not included in this study due to monomorphic traits.

### Stroke severity and functional outcome

The results of the assessments of the ten non-monomorphic SNPs of *APOD* and *SIGMAR1* against stroke severity are presented in Table 3. Analyses using Spearman’s Rho suggested that variations in one SNP, the *APOD*-encoding rs7659, is associated with NIHSS (Rho = −0.048; *P* = 0.023), while multiple logistic regression considering a NIHSS cut-off point of 16 provided an OR = 0.70; 95% CI: 0.54-0.92; *P* = 0.009. Also, an association (OR = 0.65; 95% CI: 0.46-0.91; *P* = 0.012) between the SIGMAR1 encoding rs12001648 and medium-severe stroke onset risk (NIHSS ≥ 8) was found. When a subgroup of patients aged 70 years or above was tested against the severe IS onset indicator (defined as NIHSS ≥ 16), an association between stroke severity and variants of SNP rs7659 within the *APOD* region was noticed (OR = 0.63; 95% CI: 0.45-0.88; *P* = 0.006). These results are shown in Table 4. Still, none of these tests implied any significant association when considering Bonferroni-correction. However, when considering the pathogenetic stroke main subtype CE as a subgroup for assessment we found SNP rs7659 to be significantly associated with stroke severity defined by the NIHSS > 16 cut point (OR = 0.59; 95% CI: 0.40-0.85; *P* = 0.005; results shown in Table 4).

**Table 3:** **Analysis of association between stroke severity (NIHSS) and ten** ***APOD*** **and** ***SIGMAR1*** **SNPs**

	**NIHSS score**		**NIHSS; dichotomous indicator of medium-severe (vs. mild) ischemic stroke onset (NIHSS ≥ 8)**				**NIHSS; dichotomous indicator of severe (vs. mild-medium) ischemic stroke onset (NIHSS ≥ 16)**			
			**Simple logistic regression**		**Multiple logistic regression**		**Simple logistic regression**		**Multiple logistic regression**	
**SNP**	**Estimated Spearman’s Rho**	***P*** **-value**	**OR (95% CI)**	***P*** **-value**	**OR (95% CI)**	***P*** **-value**	**OR (95% CI)**	***P*** **-value**	**OR (95% CI)**	***P*** **-value**
***SIGMAR1*** **:**										
rs11559048	0.023	0.284	1.86 (0.44-7.79)	0.399	1.74 (0.40-7.69)	0.462	--*)	--	--*)	--
rs1800866	-0.012	0.554	1.02 (0.87-1.22)	0.772	0.98 (0.81-1.17)	0.804	0.97 (0.75-1.25)	0.822	0.87 (0.64-1.17)	0.347
rs12001648	-0.044	0.040	0.77 (0.56-1.05)	0.094	0.65 (0.46-0.91)	0.012	1.01 (0.66-1.56)	0.967	0.75 (0.48-1.27)	0.283
rs7036351	0.000	0.984	1.04 (0.87-1.25)	0.670	0.99 (0.81-1.21)	0.922	0.94 (0.71-1.25)	0.664	0.84 (0.61-1.16)	0.290
rs3808873	0.011	0.627	1.04 (0.88-1.23)	0.619	1.07 (0.90-1.27)	0.457	0.79 (0.61-1.03)	0.084	0.77 (0.57-1.03)	0.079
***APOD:***										
rs76929107	0.004	0.826	0.85 (0.50-1.46)	0.555	0.74 (0.40-1.36)	0.327	0.97 (0.45-2.12)	0.948	1.17 (0.50-2.73)	0.722
rs5952	0.022	0.309	1.54 (0.38-6.18)	0.542	1.40 (0.34-5.79)	0.641	--*)	--	--*)	--
rs34697430	0.011	0.599	1.07 (0.93-1.23)	0.334	1.08 (0.94-1.25)	0.280	1.16 (0.95-1.42)	0.154	1.20 (0.96-1.50)	0.116
rs7659	-0.048	0.023	0.89 (0.77-1.04)	0.141	0.85 (0.72-1.00)	0.044	0.84 (0.66-1.06)	0.135	0.70 (0.54-0.92)	0.009
rs823510	0.013	0.538	1.03 (0.88-1.21)	0.680	1.00 (0.85-1.19)	0.965	1.06 (0.83-1.34)	0.658	0.96 (0.73-1.25)	0.736

Ischemic stroke patients from Lund Stroke Register and the Sahlgenska Academy Study on Ischaemic Stroke.

NIHSS = NIH stroke scale. *) Cannot be estimated due to monomorphism among patients with NIHSS score of 16 or above.

**Table 4:** **Detailed assessment of possible association between stroke severity (NIHSS) and SNP rs7659 within** ***APOD***

	**NIHSS score**		**NIHSS; dichotomous indicator of medium-severe (vs. mild) ischemic stroke onset (NIHSS ≥ 8)**				**NIHSS; dichotomous indicator of severe (vs. mild-medium) ischemic stroke onset (NIHSS ≥ 16)**			
**Subgroup**	**Estimated Spearman’s Rho**	***P*** **-value**	**Simple logistic regression**		**Multiple logistic regression**		**Simple logistic regression**		**Multiple logistic regression**	
			**OR (95% CI)**	***P*** **-value**	**OR (95% CI)**	***P*** **-value**	**OR (95% CI)**	***P*** **-value**	**OR (95% CI)**	***P*** **-value**
Total	-0.048	0.023	0.89 (0.77-1.04)	0.141	0.85 (0.72-1.00)	0.044	0.84 (0.66-1.06)	0.135	0.70 (0.54-0.92)	0.009
(N = 2221)			1676 controls		1618 controls		2023 controls		1947 controls	
			545 cases		495 cases		198 cases		166 cases	
Males	-0.055	0.053	0.89 (0.72-1.11)	0.293	0.85 (0.68-1.06)	0.143	0.82 (0.58-1.17)	0.273	0.75 (0.52-1.10)	0.144
(N = 1232)			971 controls		939 controls		1146 controls		1105 controls	
			261 cases		243 cases		86 cases		77 cases	
Females	-0.040	0.209	0.90 (0.73-1.11)	0.311	0.85 (0.68-1.07)	0.168	0.85 (0.62-1.16)	0.311	0.66 (0.46-0.96)	0.028
(N = 989)			705 controls		679 controls		877 controls		842 controls	
			284 cases		252 cases		112 cases		89 cases	
LSR	-0.054	0.026	0.87 (0.73-1.03)	0.113	0.81 (0.67-0.98)	0.026	0.79 (0.61-1.03)	0.081	0.65 (0.48-0.88)	0.005
(N = 1683)			1263 controls		1204 controls		1523 controls		1450 controls	
			420 cases		377 cases		160 cases		133 cases	
SAHLSIS	-0.032	0.454	0.97 (0.71-1.33)	0.858	0.94 (0.68-1.30)	0.702	1.03 (0.62-1.73)	0.902	0.86 (0.48-1.53)	0.605
(N = 538)			413 controls		412 controls		500 controls		497 controls	
			125 cases		118 cases		38 cases		33 cases	
Age < 70	-0.066	0.029	0.89 (0.70-1.13)	0.333	0.85 (0.67-1.09)	0.201	0.98 (0.66-1.47)	0.940	0.81 (0.51-1.27)	0.348
(N = 1084)			868 controls		853 controls		1021 controls		1001 controls	
			216 cases		202 cases		63 cases		54 cases	
Age ≥ 70	-0.042	0.156	0.88 (0.72-1.08)	0.215	0.82 (0.66-1.02)	0.080	0.76 (0.57-1.01)	0.060	0.63 (0.45-0.88)	0.006
(N = 1137)			808 controls		750 controls		1002 controls		946 controls	
			329 cases		266 cases		135 cases		112 cases	
LVD	-0.008	0.918	1.14 (0.70-1.87)	0.597	0.97 (0.57-1.66)	0.924	1.20 (0.54-2.67)	0.651	0.53 (0.17-1.65)	0.272
(N = 185)			133 controls		128 controls		170 controls		163 controls	
			52 cases		45 cases		15 cases		10 cases	
SVD	-0.019	0.667	0.77 (0.46-1.30)	0.329	0.81 (0.47-1.38)	0.432	N/A		N/A	
(N = 500)			461 controls		452 controls					
			39 cases		38 cases					
CE	-0.076	0.065	0.89 (0.70-1.15)	0.367	0.78 (0.59-1.03)	0.081	0.72 (0.52-1.00)	0.051	0.59 (0.40-0.85)	0.005
(N = 595)			364 controls		338 controls		484 controls		448 controls	
			231 cases		204 cases		111 cases		94 cases	

Ischemic stroke patients from Lund Stroke Register (LSR) and the Sahlgenska Academy Study on Ischemic Stroke (SAHLSIS).

NIHSS = NIH stroke scale. Multiple logistic regression models are controlling for covariates age, gender, diabetes mellitus, hypertension and current smoking when analyzing the entire sample as well as separate study groups and age groups. When analyzing males and females separately the covariate gender is omitted from these multivariable models. Pathogenetic ischemic stroke subtype: LVD = Large vessel disease; SVD = Small vessel disease; CE = Cardioembolic stroke. N/A = not applicable due to absence of sampling units with NIHSS ≥ 16.

None of the ten non-monomorphic SNPs significantly affected functional outcome after stroke (Table 5).

**Table 5:** **Analysis of association between outcome after stroke (shown by modified Rankin Scale, mRS) and ten** ***APOD*** **and** ***SIGMAR1*** **SNPs**

	**Deceased patients** ***are not*** **included in mRS:**						**Deceased patients** ***are*** **included in mRS:**					
	**Ordinal score:**		**Dichotomous indicator for dependence of support:**				**Ordinal score:**		**Dichotomous indicator for dependence of support:**			
**SNP**	**Estimated Spearman’s Rho**	***P*** **-value**	**Simple logistic regression**		**Multiple logistic regression**		**Estimated Spearman’s Rho**	***P*** **-value**	**Simple logistic regression**		**Multiple logistic regression**	
			**OR (95% CI)**	***P*** **-value**	**OR (95% CI)**	***P*** **-value**			**OR (95% CI)**	***P*** **-value**	**OR (95% CI)**	***P*** **-value**
***SIGMAR1*** **:**												
rs11559048	0.045	0.081	3.35 (0.80-14.1)	0.099	2.75 (0.49-15.3)	0.249	0.026	0.286	2.64 (0.63-11.1)	0.185	2.29 (0.38-13.7)	0.364
rs1800866	0.011	0.655	1.13 (0.93-1.37)	0.231	1.10 (0.88-1.36)	0.404	0.004	0.872	1.08 (0.90-1.30)	0.402	1.06 (0.85-1.31)	0.612
rs12001648	-0.012	0.631	0.94 (0.67-1.32)	0.712	0.77 (0.53-1.11)	0.165	-0.001	0.963	0.98 (0.72-1.34)	0.892	0.75 (0.53-1.07)	0.115
rs7036351	0.006	0.817	1.10 (0.89-1.33)	0.390	1.03 (0.83-1.29)	0.781	-0.007	0.772	1.04 (0.86-1.25)	0.722	0.97 (0.79-1.21)	0.810
rs3808873	0.021	0.436	1.08 (0.90-1.29)	0.392	1.11 (0.91-1.35)	0.290	0.012	0.636	1.05 (0.89-1.24)	0.598	1.07 (0.89-1.30)	0.457
***APOD*** **:**												
rs76929107	0.002	0.947	0.98 (0.54-1.78)	0.943	0.95 (0.49-1.82)	0.868	-0.013	0.582	0.87 (0.49-1.54)	0.623	0.92 (0.48-1.73)	0.790
rs5952	0.016	0.520	1.98 (0.28-14.1)	0.494	2.90 (0.32-26.7)	0.346	0.025	0.306	2.35 (0.39-14.1)	0.350	3.09 (0.35-27.5)	0.312
rs34697430	-0.019	0.460	0.91 (0.78-1.06)	0.221	0.91 (0.77-1.07)	0.243	0.002	0.946	0.96 (0.83-1.10)	0.514	0.92 (0.79-1.08)	0.295
rs7659	0.002	0.929	1.03 (0.88-1.22)	0.684	0.98 (0.82-1.17)	0.832	-0.005	0.846	1.02 (0.87-1.18)	0.842	0.96 (0.81-1.14)	0.665
rs823510	0.012	0.653	1.01 (0.85-1.21)	0.882	1.02 (0.84-1.23)	0.871	-0.008	0.742	0.97 (0.82-1.14)	0.696	0.97 (0.81-1.17)	0.764

Ischemic stroke patients from Lund Stroke Register and the Sahlgenska Academy Study on Ischemic Stroke.

mRS = modified Rankin Scale. Ordinal score comprises distinguishable categories 0–2, 3, 4, 5 and, if deceased patients are included, also category 6. Correspondingly, dichotomous indicator comprises categories 0–2 as control subjects and category 3 or above as cases.

## Discussion

With this large study sample comprising a total of 3081 IS patients we were able to perform analyses aimed to find possible impact of selected polymorphisms encoding for *APOD* and *SIGMAR1* on (1) stroke severity and (2) stroke outcome, respectively. By adding 1595 control subjects not suffering any stroke onset from the same geographical areas and with the same age and gender distribution as the IS patients, we have also been able to examine possible effect of these polymorphisms on IS risk.

The conclusions from the non-parametric Spearman correlation analyses (NIHSS and mRS-scores, respectively, against SNP variations) were based upon *P*-values obtained by using a “conservative” approach providing high adequacy at the cost of some statistical power loss. The transformation of these numerical variables into dichotomized indicators (coded 1 or 0) also caused information loss. On the other hand this enabled us to focus on possible threshold effects when examining e.g. the genetic effect on IS severity (by using NIHSS cutpoints 0–7 vs. 8 or above, or 0–15 vs. 16 or above).

The *SIGMAR1* region on chromosome 9p13 displays two polymorphisms that have a strong influence in CNS disease, namely rs1799729 (GC-241-240TT) and rs1800866 (Gln2Pro) that show LD forming haplotypes GC-Q and TT-P [39-41]. The rs1799729 is found in the proximal promoter region while rs1800866 is present in the first exon. Only rs1800866 was analysed in our study but these two SNPs are closely related (nearly in complete LD with r^2^ = 0.98) and have been reported to be associated with neuroprotection and risk of AD [41], and also risk of depression and alcoholism [38,40]. The polymorphism Gln2Pro is located in the amino acid sequence motif MQWAVGRR [53] at the N-terminal part of the protein, which is an endoplasmatic binding region. Hence, a mutation could affect trafficking of SIGMAR1 associated processes, which have been implicated in rodent models of stroke [37]. However; we could not find any association between the *SIGMAR1* polymorphisms and stroke risk, severity or recovery. The significance of the weak association of rs12001648 needs further investigation.

Although rigorous statistical analysis did not provide clear evidence of an association between the *APOD* SNPs and stroke risk, severity or outcome, the possible genetic influence of polymorphism rs7659 is interesting and potentially relevant. Rs7659 is located in the 3′UTR of *APOD*, and a functional variant in this area might influence the transcription of the gene or mRNA splicing. Indeed, this SNP appears to be positioned at a putative binding site for the human splicing factor SR SC35 [54]. Also, it is previously shown that rs7659 may be associated with early onset AD within the subgroup of patients lacking the APOEε4 allele [28] and with long term clinical outcome in schizophrenics [54]. Moreover, the *APOD* gene is localized on chromosome 3q2.2-qter in close proximity to the 3q25-26 region linked to AD [24]. Hence, taking into consideration the association between rs7659 to other CNS disease and our finding of a possible association of rs7659 with stroke risk and stroke severity, particularly among the elderly, this strongly encourages further studies of rs7659.

Possible occurrence of false positive *P*-values was supressed by Bonferroni correction. False negative results cannot be detected since we do not know the infinite population behind our predetermined study sample. By performing a post hoc power analysis including stroke severity (from NIHSS case–control calculations) we found rather modest statistical powers (between 5% and 41%), indicating a weak incentive for replicative studies to find an association between the selected SNPs and stroke severity (and even outcome, defined by the mRS nomenclature).

## Conclusion

In this first attempt to study if stroke repair mechanisms linked to certain regions within the *APOD* and *SIGMAR1* genes may also affect recovery from stroke and severity of stroke, we performed a candidate gene study including twelve SNPs from these two genetic regions.

Our data suggest that the rs7659 SNP within the *APOD* gene could be associated with risk for stroke and stroke severity at stroke onset. This mutation may decrease the levels of APOD and thereby diminish its protective cell signalling and antioxidant action. However, these associations showed only modest statistical significance, suggesting that our study may be underpowered despite the large sample size.
